# Tenth Annual Session of the Indiana State Medical Society

**Published:** 1859-07

**Authors:** Nathan Johnson, F. S. Newcomer, T. Parvin

**Affiliations:** President; Secretary; Secretary


### TENTH ANNUAL SESSION OF THE INDIANA STATE MEDICAL SOCIETY.

TUESDAY MORNING, MAY 17.

The Society convened at half-past ten o'clock, in the hall of the House of Representatives, Dr. Bullard presiding.

Dr. Dunlap, the chairman of the Executive Committee, reported the order of business, which was adopted.

Adjourned till two o'clock P.M.

AFTERNOON SESSION.

Dr. Bullard in the chair. Minutes read and adopted.

Dr. Dunlap moved that a committee be appointed to conduct the President elect to the chair. Drs. Dunlap and Mears were appointed said committee.

Dr. Johnson was welcomed to the chair by the retiring President.

Dr. Davidson moved that a committee of three be appointed to select some member to deliver an address in this place this evening.

Drs. David, Brower and Fishback were appointed.

Dr. T. Parvin was appointed Secretary *pro tempore*.

The President appointed a committee on admissions—Drs. Bullard, Kersey and Meeker.

The committee appointed to report some member to deliver an address before the Society, reported the name of Dr. Bullard, the address to be delivered at the hall of the House of Representatives, at a quarter before eight o'clock this evening.

Committee upon admissions reported the following names:

W. H. Cyrus, Allisonville.

Nathan Mendenhall, Plainfield.

C. F. Elder, Knightstown.

Isaac S. Collings, Cicero.

M. J. Lynch, Indianapolis.

J. B. Davis, Indianapolis.

H. Winton, North Manchester, Wabash county.

Dr. J. McFadden Gasten, of South Carolina, was elected honorary member.

Dr. Bullard offered the report for the Treasurer, Dr. Parry. Received.

The Chairman of the Committee on Publication reported verbally, and asked to be discharged.

Committee on Finance asked further time to report. Granted.

Committee on Ethics—no report.

Committee on Diseases of Brain—no report.

Committee on Practice of Medicine—Dr. Kitchen read a report from Dr. Woodworth, chairman of the committee. Reported to Committee on Publication.

Committee on Surgery—The Chairman, Dr. Spencer, offered his report through the Secretaries. Report read, received and referred to Committee on Publication.

Committee on Obstetrics, Dr. Ayres, chairman, offered his report through Dr. Kitchen. Report read, and referred to Committee on Publication.

Committee on Medical Education, Dr. Smith, chairman, asked that it might be made the special order for to-morrow, at ten o'clock A. M.

Committee on Materia Medica—no report.

Adjourned to quarter before eight this evening.

EVENING SESSION.

Society met pursuant to adjournment, the President in the chair.

Dr. Dunlap moved that a committee on nominations, to consist of five, be appointed by the chair.

The Chair appointed the following committee:

Drs. Dunlap, New, Ritter, Meeker, and Kitchen.

Dr. Bullard was then called upon to deliver his address.

A copy of the address was requested for publication.

Adjourned to meet at nine o'clock Wednesday morning.

SECOND DAY—WEDNESDAY MORNING, MAY 18.

Society met at ten o'clock, pursuant to adjournment, the President in the Chair.

A resolution making the election of officers *viva voce*, instead of by ballot, lying over from last year, was, on motion of Dr. Woodburn, adopted.

The Committee on Nominations made the following report:

President—David Hutchinson, M.D.

Vice-Presidents—John Sloan, M.D.; R. M. O'Ferral, M.D.; J. S. McClelland, M.D.; R. E. Houghton, M.D.

Corresponding Secretary—George M. Darrach, M.D.

Recording Secretaries—F. S. Newcomer, M.D.; T. Parvin, M.D.

Delegates to the American Medical Association—Drs. Chas. Fishback, S. G. Collier, Wm. Davidson, M. M. Latta, L. Humphreys, C. West, T. W. Fry, H. Winton, W. C. Thompson, R. De Pew, G. S. Freeman, Wm. Lomax, Horace Coleman, W. H. Wishard, M. H. Wright, P. H. Jameson, T. W. Taylor, Henry Cox, S. B. Butler, Wm. Readea, S. D. Day, A. M. Vickery and H. D. Henderson.

Delegates to the Ohio State Medical Society—Drs. Charles Parry and T. Parvin.

*Special Committees.*

Progress of Medicine—J. H. Brower, M.D.

Surgery—D. Meeker, M.D.

Obstetrics—Wm. Davidson, M.D.

Medical Education—Charles Fishback, M.D.

New Remedies—W. T. S. Cornett, M.D.

Operations for the Relief of Defective Vision—T. Parvin, M.D.

Continued Fevers—P. H. Jameson, M.D.

Microscopic Investigation—C. West, M.D.

Report adopted.

Dr. Bullard, Chairman of the Committee on Finance, reported the annual assessment to be two dollars. Adopted.

Dr. George Free, of Cincinnati, Ohio, was elected an honorary member.

Dr. Meeker, Chairman of the Committee on False Joints, which was continued from last year, read his report. This report was referred to the Committee on Publication.

The hour for the special order of the day having been announced by the Chair, Dr. Fishback, from the Committee on Medical Education, offered his report. Referred to Committee on Publication.

The resolutions accompanying the report were then taken up *seriatim*, and after several amendments, adopted as follows:

*Resolved*, That, concurring in the general principles of the foregoing report, and for the purpose of developing and diffusing a correct public sentiment both in and out of the profession in reference, and for the further purpose of securing concert and harmony of action in some definite proposal to the next Legislature for its official action in behalf of the public good, this Society will hold an extra meeting in this city one day before the next annual meeting, for further discussion of the subjects embraced therein. Second, that local Medical Societies throughout the State, and all members of this Society, be urged to agitate the subject in their respective localities. Third, that a Committee of Arrangements and Correspondence be appointed to make all needful preparations for the proposed extra meeting.

Dr. Dunlap was requested to read his paper on "The Plurality of the Races of Men, and their Destiny," a copy of which was requested for publication.

Adjourned to half-past one o'clock P.M.

AFTERNOON SESSION.

The Society met pursuant to adjournment, the President in the chair.

The Committee on "the Treatment of Syphilitic Diseases without the use of Mercury," reported by Dr. Haughton, chairman. Read, and referred to the Committee on Publication.

A paper on Microscopy, sent by Dr. West, was read by Dr. Hutchinson, and referred to the Committee on Publication.

[At this point the Society took a recess of an hour, to accept of the hospitalities of our ex-President, Dr. Bullard.]

The President appointed the following standing committees:

*Executive.*—Drs. P. H. Jameson, C. Brown, J. M. Gaston, T. B. Harvey and J. J. Wright.

*Finance.*—Drs. T. Bullard, W. R. Winton, B. S. Woodworth, M. H. Harding, and G. M. Darrach.

*Publication.*—Drs. T. Parvin, F. S. Newcomer, J. H. Woodburn, J. M. Kitchen, and R. E. Haughton.

*Ethics.*—Drs. H. P. Ayres, C. Parry, R. D. Mauzy, Ben. Newland and C. Bowman.

*Committee of Correspondence and Arrangements for Extra Meeting.*—Drs. M. H. Wright, J. M. Kitchen, and C. Fishback.

Dr. Brower offered the following preamble and resolutions:

WHEREAS, By a resolution of the American Medical Association, at its late meeting, the subject of the establishment of asylums for the treatment and cure, and by adequate remedies and moral agencies, of confirmed inebriates, was commended to the notice of the several State and County Medical Societies, and

WHEREAS, This Society recognizes in this movement, now in progress, especially in the State of New York, a principle, which, if effectually carried out, may result in the permanent cure and restoration to the useful pursuits of life, many of the unfortunate victims of this form of combined physical and mental disease, and thus carry out one of the most benevolent movements of the age; therefore,

*Resolved,* That a committee of one be appointed, whose duty it shall be during the recess of this Society, to collect from authentic sources, such information and statistics upon the subject as may enable its members more fully to appreciate its importance and carry out its object.

Dr. Brower was appointed said committee.

Dr. Fishback offered the following resolution, which was adopted:

*Resolved,* That a committee be appointed on criminal abortion, to collect statistics in this State embracing the number of attempts, in married and unmarried—the means used for producing—and mode of action of each kind of means.

The President appointed Dr. Fishback chairman.

Dr. Harvey read a report of a case, which was referred to the Committee on Publication.

Dr. Harvey moved that a delegation of three be appointed to attend the Illinois State Medical Society.

The President appointed Drs. Harvey, Fishback and R. M. O'Ferrall.

Dr. H. Winton read a report of a case, which was referred to Committee on Publication.

Adjourned to half-past seven o'clock P.M.

EVENING SESSION.

The Society met pursuant to adjournment, the President in the chair. Minutes read and approved.

Dr. Davidson read a paper on Scorbutus. Referred to the Committee on Publication.

A vote of thanks was tendered the Librarian for the use of the Hall.

Dr. Winton reported several surgical cases. Referred to the Committee on Publication.

The following members were present: Drs. T. Bullard, L. Dunlap, J. H. Woodburn, C. Brown, J. M. Kitchen, T. Parvin, Wm. Davidson, C. Fishback, D. Meeker, H. D. Henderson, G. W. Mears, R. M. Wellman, T. B. Harvey, L. Ritter, R. C. Moore, J. N. Green, Henry Cox, J. J. Rooker, J. S. McClelland, J. H. Bromer, D. Hutchenson, Nathan Johnson, V. Kersey, Wm. R. Smith, G. W. New, R. E. Haughton, W. C. Thompson, M. H. Wright, Wm. H. Green, S. A. Kennedy, John Lewis, F. M. Mothershead, J. H. Jameson, T. M. Stevens, L. Todd, J. M. Gaston, D. Funkhouser, G. M. Darrick, J. S. Bobbs, C. Parry, William Reader, S. G. Wallace, S. G. Collier, A. J. Mullen, B. Bartholomew, J. S. Comingore, S. M. Vickney, S. J. Boynton.

Adjourned to meet third Tuesday in May, 1860.

NATHAN JOHNSON, *President.*

F. S. NEWCOMER,<br>T. PARVIN, } *Secretaries.*

### MISCELLANEOUS MEDICAL INTELLIGENCE.

*Suspension of a Clinical School.*—The *Peninsular and Independent* announces the suspension of the Clinical School connected with the medical department of the University of Michigan.

---

*New Colleges.*—The *American Medical Gazette* states that at the close of his winter's clinical course, at Bellevue Hospital, Dr. James R. Wood announced a new medical school in connection with that institution as in embryo.

The physicions of Alabama are making efforts to establish a medical school in Mobile, Alabama. D.

---

*Appointment and Resignation of Medical Professors.*—Dr. George B. Wood has resigned the posts he has filled for many years as Professor of Theory and Practice in the University of Pennsylvania, to take effect at the close of the next lecture term, and of Physician to the Pennsylvania Hospital, where Dr. F. G. Smith has already succeeded him.

The Faculty of the Medical Department of Pennsylvania College have resigned simultaneously, and the Faculty of the Philadelphia Medical College have been elected to occupy their places. The causes of this summary change have not been made public.

---

*New York Colleges.*—Drs. C. R. Gilman and B. F. Barker have resigned their respective chairs as Professors of Obstetrics and the Diseases of Women and Children, the former in the College of Physicians and Surgeons, and the latter in the New York Medical College. Dr. Geo. T. Elliott, jr., has been appointed to the vacancy occasioned by Prof. Gilman's resignation, and Dr. E. R. Peaslee to fill Prof. Barker's chair.

Dr. Markoe, clinical lecturer in New York Hospital, has been appointed Adjunct Professor of Surgery with Prof. Willard Parker.

Dr. Austin Flint, jr., the able editor of the *Buffalo Medical Journal*, has been appointed to the chair of Physiology and Microscopic Anatomy in the University of Buffalo, and Dr. S. Eastman to the chair of Anatomy.

---

*Medical Journals.*—The *Montreal Medical Chronicle* has ceased to be issued. Its editors say the action of the new Canada postage law would render its publication an expense to its publishers.

The *Maine Medical Journal* has not been sustained by the physicians of that State, and consequently its editor refuses to issue it another year.

The *Buffalo Medical Journal* will hereafter be edited by its former editor, Dr. Flint, jr., at New York, and continue to be published at Buffalo. D.

